# Safety and tissue yield for percutaneous native kidney biopsy according to practitioner and ultrasound technique

**DOI:** 10.1186/1471-2369-15-96

**Published:** 2014-06-23

**Authors:** Sungjin Chung, Eun Sil Koh, Sung Jun Kim, Hye Eun Yoon, Cheol Whee Park, Yoon Sik Chang, Seok Joon Shin

**Affiliations:** 1 Department of Internal Medicine, College of Medicine, The Catholic University of Korea, 222 Banpo-daero, Seoul 137-701, Republic of Korea; 2 Division of Nephrology, The Catholic University of Korea Yeouido St. Mary’s Hospital, 10, 63-ro, Yeongdeungpo-gu, Seoul 150-713, Republic of Korea; 3 Division of Nephrology, The Catholic University of Korea Incheon St. Mary’s Hospital, 56, Dongsu-ro, Bupyeong-gu, Incheon 403-720, Republic of Korea; 4 Division of Nephrology, The Catholic University of Korea Seoul St. Mary’s Hospital, 222 Banpo-daero, Seoul 137-701, Republic of Korea

**Keywords:** Percutaneous renal biopsy, Ultrasound, Outcome, Safety

## Abstract

**Background:**

Although percutaneous renal biopsy remains an essential tool in the diagnosis and treatment of renal diseases, in recent times the traditional procedure of nephrologists has been performed by non-nephrologists rather than nephrologists at many institutions. The present study assessed the safety and adequacy of tissue yield during percutaneous renal biopsy according to practitioners and techniques based on ultrasound.

**Methods:**

This study included 658 native renal biopsies performed from 2005 to 2010 at a single centre. The biopsies were performed by nephrologists or expert ultrasound radiologists using the ultrasound-marked blind or real-time ultrasound-guided techniques.

**Results:**

A total of 271 ultrasound-marked blind biopsies were performed by nephrologists, 170 real-time ultrasound-guided biopsies were performed by nephrologists, and 217 real-time ultrasound-guided biopsies were performed by radiologists during the study period. No differences in post-biopsy complications such as haematoma, need for transfusion and intervention, gross haematuria, pain, or infection were observed among groups. Glomerular numbers of renal specimens from biopsies performed by nephrologists without reference to any technique were higher than those obtained from real-time ultrasound-guided biopsies performed by expert ultrasound radiologists.

**Conclusions:**

Percutaneous renal biopsy performed by nephrologists was not inferior to that performed by expert ultrasound radiologists as related to specimen yield and post-biopsy complications.

## Background

Since the first report by Iversen and Brun in 1951, percutaneous renal biopsy has made many contributions to the diagnosis and management of patients with renal diseases [1,2]. The impact of renal biopsy has been established, and this procedure influences the appearance of nephrology as a specialty [3]. Indeed, Brun, a pioneer who first successfully performed a major series of renal biopsies, was one of the first nephrologists [3].

In recent times percutaneous renal biopsies have been taken over by non-nephrologists, particularly radiologists, at many institutions. Approximately 40% of biopsies done in the United States in 1993 were performed by radiologists [3]. A survey that determined who is performing kidney biopsies at university hospitals in Korea showed that only 26.1% of renal biopsies are performed by nephrologists and 42.9% of renal biopsies are performed by radiologists [4]. Why has the initiative for percutaneous renal biopsy gone to radiologists? The probable reason is that nephrologists have lost interest in the renal biopsy procedure possibly because they became occupied elsewhere or are reluctant to take the procedure-related risk. Many nephrologists do not feel the need to personally perform percutaneous renal biopsies as there is a need for nephrologists in the field of renal replacement therapy such as haemodialysis, peritoneal dialysis and kidney transplantation [3]. Furthermore, some nephrologists have preconceived notions that they are inferior to radiologists in terms of access or skills with imaging devices such as ultrasound (US) [4]. Young nephrologists inexperienced with the biopsy technique and US may be reluctant to become involved with an invasive procedure due to fear of post-biopsy complications [4].

The aim of this study was to show whether renal biopsy performed by nephrologists is as safe and effective as that performed by radiologists. We compared the safety and adequacy of glomerular yield from percutaneous renal biopsy between cases performed by nephrologists and those performed by radiologists. Some renal biopsies had been performed by nephrologists at our teaching hospital, whereas others had been performed by radiologists. In cases performed by nephrologists, percutaneous renal biopsy was done under real-time US-guidance or blindly according to their individual preference.

## Methods

### Study population

This analysis included all adult patients who underwent percutaneous native renal biopsies at The Catholic University of Korea St. Mary’s Hospital during the 6-year period from January 2005 to December 2010. During the study period, renal biopsies were never performed in the patients with polycystic kidneys, a single kidney, and those who were pregnant. Biopsies of a transplanted kidney or right kidney and transjugular renal biopsies were excluded. In addition, 1-day case renal biopsies without hospitalisation were not included. At our institution, it was for attending nephrologists to decide how to perform renal biopsies of their assigned patients in the light of their own preference for the procedure. A total of 658 native renal biopsies were performed. Among them, 441 were performed by nephrologists and 217 by radiologists (Group III). The 441 cases performed by nephrologists included 271 cases of blind biopsy (Group I) and 170 cases of a real-time US-guided procedure (Group II). Haemoglobin, haematocrit, platelet count and international normalized ratio (INR) were measured prior to biopsy to minimize the risk of biopsy-related bleeding. Levels of serum creatinine, blood urea nitrogen (BUN) and 24-hour urinary protein were also included at the time biopsy. Kidney function was determined using an abbreviated equation developed using data from the Modification of Diet in Renal Disease study to estimate glomerular filtration rate [5]. Aspirin or other anti-platelet agents were withdrawn for at least 7 days before the procedure. In cases of hypertension under treatment, the biopsy was performed when blood pressure < 140/90 mmHg was achieved. Patients lay in a bed on their back for at least 8 hours following the biopsy. Informed consent was mandatory and was obtained from all patients. This retrospective study was approved by the Institutional Review Board of The Catholic University of Korea St. Mary’s Hospital (No. OC11RISI0037).

### Biopsy technique

All patients had prior diagnostic US or computed tomography (CT) to exclude patients with a structural cause of renal insufficiency and to avoid biopsy of single or small kidneys. The biopsy was performed in the prone position with patients lying with their abdomen on a firm pillow. The lower pole of the left kidney was located by US. After disinfecting the skin, lidocaine was injected subcutaneously, and subsequently deeper towards the kidney. A small incision was made to facilitate introducing the biopsy needle.

In cases performed by US-marked blind renal biopsy, a nephrologist advanced the probe needle perpendicularly through the skin after marking the skin over the lower pole of the left kidney under US guidance. The biopsy needle was properly placed in the renal capsule by feeling the resistance of solid tissue or by observing respiratory excursion of the needle. After estimating depth from the skin, the probe needle was withdrawn, and the 14 G needle of the automated biopsy gun (C. R. Bard, Inc., Murray Hill, NJ, USA) was advanced to the previously estimated depth. When patients were asked to hold their breath, the gun was fired, and the needle was removed.

The procedures were similar between nephrologists and radiologists for real-time US-guided renal biopsy. After local anaesthesia, a practitioner advanced the automated biopsy needle to the lower pole of the left kidney capsule under renal-time US guidance, and then the biopsy gun with 14 G needle was fired. Nephrologists conducted the biopsy procedure holding the US transducer in the left hand and the biopsy gun in the right hand, while radiologists performed the biopsy with or without using the disinfected biopsy needle bracket that attached to the transducer.

In all cases performed by nephrologists, a first- or second-year fellow conducted the entire procedure. When the renal biopsy was requested by the Department of Radiology, all cases were performed with a real-time US-guided procedure by attending radiologists whose major area of expertise was abdominal radiology and US. All practitioners, both nephrologists and radiologist, have been requested to obtain two specimens of the kidney. In all renal biopsies performed by radiologists, a nephrology fellow has observed the whole process of the procedure and checked the obtained specimens with naked eye. When the renal specimen was deemed inadequate by gross visual inspection, the nephrology fellow made a request to the radiologist for additional pass. Consequently, the average of two renal specimens was obtained. After the procedure, the specimens were hand-delivered by nephrology residents to the Department of Pathology for histologic evaluation.

### Biopsy safety and yield

Post-biopsy US imaging was obtained to identify immediate bleeding complications. After returning to the hospital ward, all patients lay in bed on their backs for a 24 hour observation time, and blood pressure and pulse rate were monitored frequently. During this time, close observation for gross haematuria, flank and abdominal pain, hypotension, and fever was performed. If the patient showed unstable vital and clinical signs such as hypotension, tachycardia or abdominal pain, an immediate CT scan or US of the abdomen was obtained. Blood transfusions or radiological intervention including renal artery embolization were provided when clinically indicated. Blood haemoglobin and haematocrit were measured 24 hours after the biopsy. Each patient had an outpatient follow-up visit with their nephrologist within 2 weeks of the kidney biopsy. All patients were told to visit the outpatient clinic during the day or emergency room at night if they suffered any newly developed symptoms including pain or fever after discharge from the hospital. Readmission to the hospital was allowed when clinically indicated.

Glomerular yield was expressed as the total number of glomeruli present in the specimen. This included the number of all glomeruli in samples as assessed by light microscopy, immunofluorescence, and electron microscopy.

### Statistical analysis

Data are presented as mean ± standard deviation or range for continuous variables. Categorical variables are presented as frequency counts and percentages. Differences between groups were detected by analysis of variance with Bonferrroni’s correction. Multiple linear regression was performed to identify the variables that made an important contribution to number of glomeruli from renal specimens and to adjust for confounding variables. We examined the association between clinical and laboratory factors and prodedure-related complications using multiple logistic regression analysis. The variables included in this analysis were sex, age, body mass index (BMI), presence of diabetes, hypertension, systemic lupus erythematosus (SLE) and chronic liver disease, blood pressure, pre-biopsy haemoglobin, platelet count, proteinuria and estimated glomerular filtration rate (eGFR). A p-value < 0.05 was considered significant.

## Results

### Baseline characteristics of patients

The mean age of all patients was 37 years, and 4.7% of the patients were > 65 years. Men comprised 52.6% of all biopsy patients. Hypertension, diabetes, SLE, and chronic liver disease were found in 20.8%, 4.1%, 10.8% and 3.5% of all patients respectively. The mean BMI of all patients was 23.1 ± 3.6 kg/m^2^. When patient demographics in the three renal biopsy groups were compared, the groups were similar in age, sex, weight, height, BMI, and blood pressure (Table 1). The prevalence of diabetes was significantly higher in Group II when compared with that of Group I. In addition, the prevalence of SLE was significantly higher in Group II when compared with that of Group III.

**Table 1:** Clinical features of the study groups

	**Group I (n = 271)**	**Group II (n = 170)**	**Group III (n = 217)**
Age, years	35.8 ± 14.4	38.6 ± 15.0	36.4 ± 15.9
Male	145 (53.5)	89 (52.4)	112 (51.6)
Weight, kg	62.4 ± 11.2	63.1 ± 13.2	63.9 ± 12.5
Height, cm	165.0 ± 8.8	165.0 ± 9.6	164.5 ± 10.0
BMI, kg/m^2^	22.9 ± 3.3	23.0 ± 3.7	23.5 ± 3.8
Blood pressure			
Systolic, mmHg	118 ± 15	119 ± 14	117 ± 17
Diastolic, mmHg	74 ± 11	74 ± 12	74 ± 11
Comorbid conditions			
Diabetes	5 (1.8)	14 (8.2)^a^	8 (3.8)
Hypertension	55 (20.3)	40 (23.5)	42 (19.8)
SLE	30 (11.1)	30 (17.6)^b^	11 (5.1)
Chronic liver disease	10 (3.7)	6 (3.5)	7 (3.2)

Data are presented as mean ± standard deviation or number (percentage).

^a^Group II versus Group I, p < 0.005.

^b^Group II versus Group III, p = 0.0005.

*BMI*, body mass index, *SLE*, systemic lupus erythematosus.

The overall mean baseline haemoglobin and haematocrit values were 12.7 g/dL and 37.1%, respectively, whereas the overall mean post-biopsy haemoglobin and haematocrit values were 12.4 g/dL and 36.4%, respectively. The baseline mean platelet count value of all patients was 245 × 10^3^/mm^3^, and the mean INR was 0.96. Mean BUN and serum creatinine values in all biopsy patients were 20.3 mg/dL and 1.40 mg/dL, respectively, indicating that the mean eGFR of the patients was 77.10 ± 33.37 mL/min/1.73 m^2^. The groups were similar in baseline laboratory findings including haemoglobin, haematocrit, and serum creatinine (Table 2). Platelet count was higher in Group II than that in Group III. The mean INR in Group I was significantly lower than those in Groups II and III. Although mean baseline BUN in Group I was slightly but significantly elevated compared to that of Group III, levels of serum creatinine were similar among the groups. The 24-hour urinary protein in Group I was higher than those in Groups II and III.

**Table 2:** Laboratory findings of the study groups

	**Group I (n = 271)**	**Group II (n = 170)**	**Group III (n = 217)**
Haemoglobin, g/dL			
Prebiopsy	12.7 ± 2.3	12.7 ± 3.2	12.7 ± 2.2
Postbiopsy	12.3 ± 2.3	12.3 ± 2.5	12.5 ± 2.4
ΔHaemoglobin	0.3 ± 1.2	0.4 ± 2.3	0.1 ± 1.4
Haematocrit, %			
Prebiopsy	36.8 ± 6.8	37.3 ± 6.9	37.3 ± 6.2
Postbiopsy	35.9 ± 6.6	36.6 ± 7.3	36.8 ± 6.2
ΔHaematocrit	0.5 ± 4.3	0.7 ± 2.6	0.5 ± 2.5
Platelet count, x10^3^/mm^3^	247 ± 78	234 ± 69^a^	252 ± 72
INR	0.94 ± 0.10^b^	0.97 ± 0.09	0.99 ± 0.09
BUN, mg/dL	21.9 ± 17.3^c^	20.5 ± 12.8	18.0 ± 11.7
Serum creatinine, mg/dL			
Mean ± SD	1.46 ± 1.33	1.47 ± 1.38	1.25 ± 1.17
Range	0.56-10.64	0.58-11.21	0.36-9.60
eGFR, mL/min/1.73 m^2^	74.07 ± 31.86	73.67 ± 32.41	83.56 ± 35.26^d^
24 hr Proteinuria, g/day	1.57 (0.30-4.50)^e^	0.86 (0.18-2.76)	1.43 (0.26-3.46)

Data are presented as mean ± standard deviation, range or median (interquartile interquartile, IQR).

^a^Group II versus Groups III, p = 0.0425.

^b^Group I versus Group II, p = 0.0028 and Group I versus Group III, p < 0.0001

^c^Group I versus III, p = 0.0102.

^d^Group III versus Group I, p = 0.0053 and Group III versus Group II, p = 0.0111

^e^Group I versus Group II, p = 0.0079 and Group I versus Group III, p = 0.0060.

*INR*, international normalized ratio, *BUN*, blood urea nitrogen, *SD*, standard deviation, *eGFR*, estimated glomerular filtration rate.

### Glomerular yield

Overall, the mean number of glomeruli obtained from renal specimens was 25 ± 16, and the diagnostic failure rate was 1.22%. The adequacy of renal tissue obtained by the three groups is compared in Table 3. The mean number of glomeruli in renal tissue obtained by nephrologists regardless of the method used (Groups I and II) was significantly higher than that obtained by radiologists (Group III). An adjusted multiple linear regression analysis also showed the same results (Group III versus Group I or II, p < 0.0001). No significant difference in glomerular yield was observed between Group I and Group II (p = 0.2181). The proportion of failure to make a final pathologic diagnosis was similar among all groups.

**Table 3:** Biopsy tissue adequacy of the study groups

	**Group I (n = 271)**	**Group II (n = 170)**	**Group III (n = 217)**
Number of glomeruli	26 ± 17	29 ± 17	20 ± 12^a^
Failure to diagnosis	5 (1.8)	1 (0.6)	2 (0.9)

Data are presented as mean ± standard deviation or number (percentage).

^a^Group III versus Group II or Group III, p < 0.001.

### Post-biopsy complications

The overall frequency of major bleeding complications such as development of haematoma and requirement for transfusion or intervention was 6.8%. No cases of death or nephrectomy occurred. As presented in Table 2, a significant decrease in haemoglobin or haematocrit was not seen in any of the study groups. The frequency of haematoma, transfusion, and intervention was not significant among the groups (Table 4). Gross haematuria tended to develop less often in Group II although the inter-group difference was not significant. Procedure-related infection such as pyelonephritis and readmission in relation to post-biopsy complications tended to occur more frequently in the radiologist-performed Group III, although the difference among groups failed to show a statistical difference. When multiple logistic regression analysis was performed to investigate which baseline characteristic factors were associated with post-biopsy complications in each group, there was no factor associated with an increased risk of any complication.

**Table 4:** Biopsy-related complications of the study groups

	**Group I (n = 271)**	**Group II (n = 170)**	**Group III (n = 217)**
Haematoma	10 (3.7)	10 (5.9)	12 (5.5)
Transfusion	5 (1.9)	5 (2.9)	2 (0.9)
Intervention	0 (0)	0 (0)	1 (0.5)
Gross haematuria	22 (8.1)	7 (4.1)	21 (9.8)
Pain	41 (15.1)	31 (18.2)	26 (12.1)
Infection	1 (0.4)	0 (0)	3 (1.4)
Rehospitalisation	6 (2.2)	4 (2.4)	8 (3.7)

Data are presented as number (percentage).

## Discussion

No significant differences in overall failure rate of the pathologic diagnosis or post-biopsy complications were observed between radiologists and nephrologists. Despite no influence on the overall diagnosis, the mean number of glomeruli obtained from renal biopsies performed by nephrologists was greater than that from the procedures performed by expert US radiologists.

Although complications can still occur and are mainly related to bleeding, percutaneous renal biopsy remains a relatively safe procedure with modern medical techniques [6]. Life threatening complications in the current practice of percutaneous renal biopsy occur in < 0.1% [6-8]. The needle has changed over the years as a modification that has shaped percutaneous renal biopsy into a clinically relevant, safe, and effective procedure [3,6]. With the introduction of automated spring-loaded cutting-needle biopsy guns in the 1980s, it became possible to make the procedure more effective diagnostically and safer [7]. In addition, recent advances in imaging modalities have simplified the procedure, leading to correct needle positions [9]. It is believed that the biopsy procedure conducted under real-time US imaging is safer and more successful than a blind procedure that is performed after marking the skin over the lower pole of the kidney with US [7,10]. However, our results demonstrate that the rate of biopsy-related complications following the blind procedure by nephrologists were comparable to those following real-time US-guided renal biopsies by nephrologists or radiologists, suggesting that knowledge of accurate placement of the needle in the kidney may be more important than the technique itself.

Regardless of the technique used, studies have reported adequate renal tissue sampling in 80–100% of cases [11,12]. There is a question of how many glomeruli, tubules, and vessels are necessary for an accurate diagnosis of kidney disease. When diffuse glomerular diseases such as amyloidosis and membranous glomerulonephropathy are suspected, one glomerulus is theoretically sufficient for diagnosis [12,13]. Some researchers insist on a minimum of two tissue cores [12,14], whereas others have said that at least 25 glomeruli are needed for most accurate diagnoses [13,15,16]. Considering the random distribution of focal glomerular diseases, many more glomeruli are needed. Currently, there is no agreement among institutions and investigators on the definition of tissue adequacy. Some physicians have suggested that native kidney samples should have at least 20 glomeruli to exclude focal disease processes and enable an accurate assessment of the degree glomerular involvement [9,17]. Therefore, the big challenge would be to get more glomeruli for an accurate histopathologic diagnosis while preserving tissue structure and minimizing complications.

Several studies have investigated sample adequacy and the number of glomeruli according to biopsy technique or practitioner. In some institutions, nephrologists and radiologists perform the renal biopsy procedure together as a team. In biopsies that are performed by a radiologist in concordance with a nephrologist, the nephrologist usually advances the needle to a depth deemed proper by the radiologist while the radiologist holds the transducer for real-time US guidance [18]. According to studies from such institutions, 11.6–21.6 glomeruli are obtained using real-time US guidance [18-20]. In cases that the nephrologist alone performs the whole procedure by real-time US guidance, the mean number of glomeruli obtained from specimens is 16–18 [10,21]. Real-time US guidance is always used at institutions in which the radiologist alone performs the whole biopsy procedure [9,22]. The mean number of glomeruli harvested by radiologists is 15–21 per biopsy [9,21]. In contrast, a study using the US-guided blind procedure by nephrologists showed various results from 11 glomeruli in uncounted specimens to 33 glomeruli in the average of three specimens [10,11]. In our study, we acquired > 20 glomeruli per biopsy in most samples harvested by both nephrologists and radiologists. However, all nephrologists-performed renal biopsies yielded many more glomeruli compared with those obtained by radiologists. Interestingly, the mean number of glomeruli from a renal specimen in the nephrologist-performed blind biopsies was comparable to that in the nephrologist-performed real-time US guided biopsies and was even superior to that in the radiologist-performed real-time US guided biopsies. If there is no difference in terms of procedure-related complications, it is reasonable that percutaneous renal biopsies should be operated by the practitioner showing better tissue adequacy. More glomeruli might affect or even change the pathologic diagnosis.

In recent years, there has been a marked trend toward fewer nephrologist-performed renal biopsies for reasons of liability and economics [23], and this gap has been filled by radiologists, urologists, and even rheumatologists [23-25]. Nephrologists can make real-time decisions about the adequacy of sample size given the suspected diagnosis because they have a good understanding of indications, contraindications, modifications and complications in the procedure as well as patient condition [7,26]. More importantly, losing control over renal biopsy may strike at the unconscious sense of identity of nephrology [3]. A previous report demonstrated that a nephrologist with proper training can perform procedures such as renal US, renal biopsy, placement of peritoneal dialysis catheter, catheterization for vascular access, and angioplasty safely and effectively [27]. In this study, we also found that US localisation of a kidney for biopsy was rapidly acquired by our nephrology fellows, and the outcomes of the procedure by nephrologists were at least equal to those by expert US radiologists. According to current results, all biopsies at our institution are now being conducted by nephrologists. All nephrology trainees need to learn the proper kidney biopsy technique not only because of the importance of the procedure but also because renal biopsy is one of the triggers that enable the development of nephrology [7].

Our study had some limitations. First, this was a retrospective study, which could weaken the interpretation of the results although the sample is large compared to other studies. Second, patients were not randomly assigned to the group, which meant that the choice of procedure might have been biased by the practitioner’s preference with one of the three procedures. One could postulate that more complicated and complex cases of patients were referred to radiologists for renal biopsy but we showed that clinical variables were similar among groups. Third, we examined patients visiting at a single institution, so further study is required to more fully understand the generalizability of our experiences and results to other institutions and clinicians. In fact, the current study was performed in our tertiary referral centre, whose patients would have more comorbid conditions than other institutions. However, the indications for renal biopsy would reflect what is actually present in the nephrology community at large. Finally, the number of needle passes was not recorded in every case. However, previous studies have reported that number of passes dose not affect the rate of post-biopsy complications [28,29]. Despite no documentation on the number of needle passes, we also showed that there was no significant difference in overall complications after the biopsy between nephrologists and radiologists.

## Conclusions

In summary, our analysis shows that nephrologist-performed renal biopsies resulted in a similar complication rate but greater numbers of glomeruli compared with radiologists regardless of whether the real-time US guidance or blind procedure was conducted. Because it is a delicate issue to compare the outcomes between practitioners, only one study has been reported [21], and it will be difficult to perform additional randomized trials. It is essential that nephrologists cooperate with other specialists in a variety of ways for patient care. At least, current evidence shows that trainees like nephrology fellows, if they have good outstanding of kidney structure and characteristic, can perform the renal biopsy safely and effectively, and it would be reasonable to give preference to nephrologists with respect to the ultrasound-guided percutaneous procedure.

## Competing interests

The authors declare that they have no competing interests.

## Authors’ contributions

The study was designed and implemented by SC and SJS. Data were collected and analyzed by SC, ESK, SJK, HEY, CWP, YSC and SJS. SC and SJS prepared the manuscript. SJS supervised overall project. All authors read and approved the final version of manuscript.

## Pre-publication history

The pre-publication history for this paper can be accessed here:

http://www.biomedcentral.com/1471-2369/15/96/prepub
